# Primary endocrine therapy versus surgery plus endocrine therapy for early-stage breast cancer in older women without frailty: a cost-effectiveness and value of implementation analysis

**DOI:** 10.1186/s13561-025-00668-x

**Published:** 2025-09-30

**Authors:** Yubo Wang, Li-Chia Chen, Kwok-Leung Cheung, Douglas Steinke, Sean P. Gavan

**Affiliations:** 1 Centre for Pharmacoepidemiology and Drug Safety, Division of Pharmacy and Optometry, School of Health Sciences, Faculty of Biology, Medicine and Health, The University of Manchester, 1st Floor Stopford Building, Oxford Road, Manchester, M13 9PT UK; 2 Manchester Centre for Health Economics, Division of Population Health, Health Services Research and Primary Care, School of Health Sciences, Faculty of Biology, Medicine and Health, The University of Manchester, Oxford Road, Manchester, M13 9PL UK; 3 School of Medicine, University of Nottingham, Royal Derby Hospital Centre, Uttoxeter Road, Derby, DE22 3DT UK; 4 Pharmacy Department of First Affiliated Hospital of Xinjiang Medical University, Ürümqi, China

**Keywords:** Cost-effectiveness analysis, Breast cancer, Implementation, Older women, Primary endocrine therapy

## Abstract

**Background:**

Clinical guidelines recommend surgery for early-stage breast cancer in operable patients; however, primary endocrine therapy (PET) is often used in older women aged ≥ 70. This study aimed to estimate the cost-effectiveness and value of implementation of surgery plus adjuvant endocrine therapy (ET) compared with PET for older women with early breast cancer who are fit for surgery.

**Method:**

A partitioned survival analysis model was developed using effectiveness data from the published literature (time horizon: lifetime). Health outcomes were measured as quality-adjusted life years (QALYs; EQ-5D-3L UK tariff). Direct costs were estimated from the perspective of NHS England (discount rate: 3.5%). Probabilistic sensitivity analysis and value of implementation analysis were conducted using a cost-effectiveness threshold of £20,000-£30,000 per QALY gained.

**Results:**

Surgery + ET resulted in higher QALYs (4.57) compared to PET (3.87) and higher costs (£10,628 vs. £6,102). The incremental cost-effectiveness ratio (ICER) was £6,412.62 per QALY gained, indicating that surgery + ET is cost-effective compared to PET. The value of implementation analysis showed that imperfect implementation of surgery + ET resulted in a loss of 0.12 QALYs per patient, equating to 9,267 QALYs at the population level.

**Conclusion:**

Surgery with adjuvant ET is a clinically effective and cost-effective strategy compared with PET for older women with ER + operable early-stage breast cancer. Strengthening adherence to national guidelines will improve population health outcomes and healthcare resource use. Future economic evaluations should focus on the value of management strategies for older patients unfit for surgery due to frailty or comorbidities.

**Supplementary Information:**

The online version contains supplementary material available at 10.1186/s13561-025-00668-x.

## Introduction

Due to significant advances in treatments and diagnostic capabilities, breast cancer has now transformed into a condition that often requires long-term management [1, 2]. The percentage of post-menopausal women diagnosed with early-stage breast cancer at an older age has risen consistently, particularly among those aged 70 years and above, across European countries and the United Kingdom (UK) [3]. This demographic shift, coupled with the complexity of health issues and diverse decision-making preferences among older women facing primary breast cancer, places increasing pressure on healthcare systems to deliver cost-effective care and social support [4, 5]. Primary endocrine therapy (PET) has emerged as an alternative treatment approach for older patients due to the psychological barriers and quality-of-life impact of surgical procedures [6, 7], despite clinical guidelines prioritising surgery, including breast-conserving surgery (BCS) or mastectomy (hereafter, the term surgery is used to refer to breast-conserving surgery (BCS) or mastectomy), as the first-line treatment option [8, 9].

However, there is now a substantial body of evidence to demonstrate that PET is less effective than surgical intervention, for older individuals who are physically fit to receive surgery [10, 11]. A Cochrane review and meta-analysis estimated that surgery plus endocrine therapy (ET) improved overall survival (hazard ratio: 0.86, 95% CI: 0.73 to 1.00) and progression-free survival (hazard ratio: 0.65, 95% CI: 0.53 to 0.81) compared with PET within randomised controlled trials (RCTs) for women aged 70 years and above with early-stage breast cancer [10]. More recently, a meta-analysis of RCTs and observational studies found that overall survival was worse for older women who received PET compared with surgery + ET (hazard ratio: 1.27, 95% CI: 1.05 to 1.55) [11]. In addition, a recent health economic evaluation by Holmes et al. found that surgical intervention was effective and cost-effective compared with PET for women without comorbidities between the ages of 70 and 79 [12]. While Holmes et al. established the cost-effectiveness of surgical intervention for a specific subgroup, the analysis also assumed that patients within the target population received this strategy with perfect uptake. For health policymakers, there is a growing need to understand the impact of imperfect uptake, in terms of cost and health outcomes, within this population of individuals who are eligible for surgery. The present study builds on these earlier findings to address this outstanding need for evidence explicitly.

Despite these findings and recommendations within clinical guidelines, a growing proportion of older women with early-stage breast cancer are now receiving PET as their first-line management strategy [13]. A national audit in England and Wales revealed a declining trend in primary surgery between 2014 and 2019 amongst older women with breast cancer as they advanced in age (97% for ages 50–69, 91% for ages 70–79, and 55% for ages 80 and older) [13]. In addition, 23% of older patients deemed suitable candidates for surgery underwent non-surgical intervention, for whom PET was the predominant alternative [13]. The reasons for using PET are multifaceted, with insights from qualitative research highlighting that people with breast cancer may prefer PET due to their older age, apprehensions about how surgery may impact their physical and psychological well-being, and a desire to minimise disruptions in their daily lives [14]. Similarly, breast cancer surgeons may favour PET for older women due to concerns about the potential risks of complications and the adverse impact on quality of life [15].

The gradual shift towards PET poses a potential challenge for improving outcomes amongst older people with breast cancer who are physically fit to undergo surgery and the value of healthcare delivery. All else being equal, health outcomes at a population level will be lower if fewer individuals receive a first-line surgical intervention with adjuvant ET that is more effective than PET. Moreover, the healthcare resources forgone to deliver PET could be diverted towards higher-value surgical interventions to improve the cost-effectiveness of care. Therefore, evidence is required to reinforce the current recommendations for managing early-stage breast cancer in older women and support healthcare decision-makers to improve the uptake of first-line surgery in this population where feasible.

Value of implementation analysis is a method used in health economics research to quantify the population-level health outcomes forgone when the uptake of an effective and cost-effective intervention is less than perfect [16]. A key advantage of this method is to help decision-makers understand the benefits that could be achieved by moving a greater proportion of patients within a target population towards strategies that are deemed to be cost-effective. The gap between recommendations by clinical guidelines and the observed use of PET amongst older people eligible for surgery, presents a timely need for this type of analysis to understand the corresponding impact on cost and health outcomes. The outputs from a value of implementation analysis in this context will help decision-makers understand the prevalent health burden driven by the growing rise of PET and stimulate the case for improving the uptake of more effective and cost-effective surgical intervention in line with current clinical guidelines [16]. Therefore, this study aimed to estimate the cost-effectiveness and value of implementation of surgery plus ET compared with PET for older women with early breast cancer who are fit for surgery.

## Method

### Study design

A model-based cost-effectiveness analysis and value of implementation analysis compared surgery + ET with PET for women aged 70 + with early-stage breast cancer in the UK who were suitable for surgery, without severe local spread or metastases, and with any ER status. The decision problem is summarised in Table 1. Health outcomes were measured as quality-adjusted life years (QALYs), and costs were measured from a healthcare (NHS England and Personal Social Services) perspective. A lifetime time horizon was used. Lifetime QALYs and costs were discounted at 3.5% per year. The study was reported according to the Consolidated Health Economic Evaluation Reporting Standards 2022 (CHEERS 2022) [17] (Supplementary material 1 Appendix 1).

**Table 1:** Decision problems in this cost-effectiveness analysis

Element of decision problems	Description
Population	Older women (≥ 70 years) with early-stage breast cancer (operable patients)
Intervention	Surgery, including mastectomy and breast-conserving surgery
Comparator	Primary endocrine therapy
Perspective	NHS England & Personal Social Services perspective
Measure of health outcome	Quality-adjusted life years estimated from EQ-5D-3 L UK tariff
Cost consideration	Include direct medical costs: • cost of treatment • cost of hospitalisation • cost of follow-up Exclude direct non-medical, indirect and productivity cost
Outcome	• Expected incremental cost • Expected incremental QALYs • Incremental cost-effectiveness ratio • Expected value of perfect information
Time horizon	Lifetime with a 6-month cycle length
Discount rate	Cost = 3.5%, QALYs = 3.5%
Cost-effectiveness threshold	£20,000 and £30,000 per QALY gained
Sensitivity analysis	• One-way sensitivity analysis (reported in Supplementary Material 1 Appendix 3) • Probabilistic sensitivity analysis

(Note) QALYs: Quality-adjusted life years; NHS: National Health Service

### Model structure

**Fig. 1:**
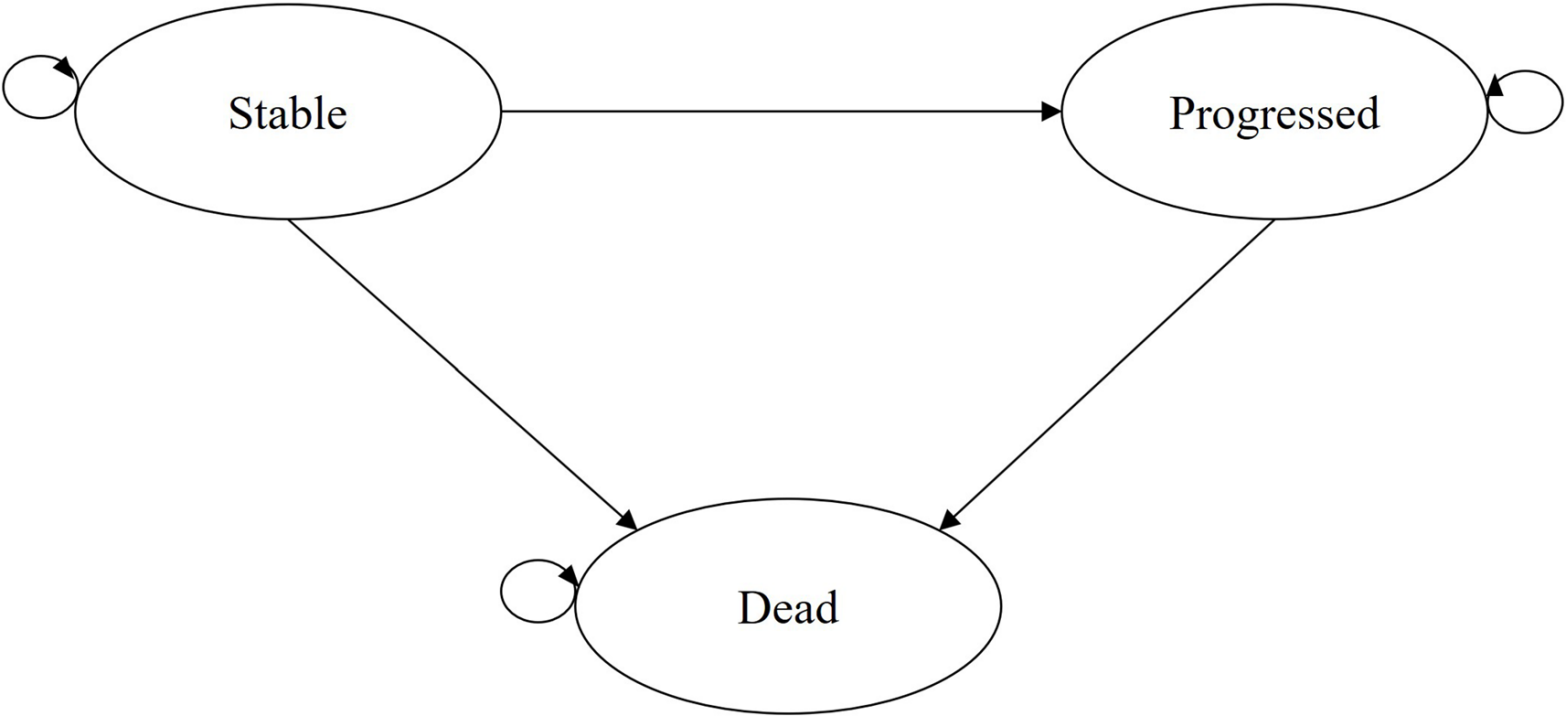
Model structure

The model structure was a partitioned survival analysis (Fig. 1) using three health states (stable, progressed, and dead). The model was built in Microsoft Excel (Version 2507 Build 16.0.19029.20136). The structure was informed by a systematic review of cost-effectiveness analyses for older women with breast cancer [18], the cost-effectiveness analysis by Holmes et al. [12], and input from clinical experts which concluded that the three-state partitioned survival analysis was appropriate to reflect the natural history of older people with primary breast cancer. The model was validated according to the AdViSHE tool (Supplementary material 2). The cycle length was 6-months (half-cycle correction was applied). The input parameters are reported in Table 2.

**Table 2:** Input parameters for the Markov model

Parameters	Deterministic analysis			Probabilistic analysis		Data source
	Value	Range (95% CI)		Distribution	Parameters	
**Transition probabilities**						
**Surgery**						
From disease-free to death	HR: 0.86	(0.73, 1.00)		Lognormal (Mean, SD)	0.86, 0.07	[10]
From disease-free to progressed disease	HR: 0.65	(0.53, 0.81)		Lognormal (Mean, SD)	0.65, 0.07	[10]
**PET**						
From disease-free to death	Log-logistic shape: 1.72 location: 0.41	shape (1.60, 1.85) scale (0.36, 0.48)		Multivariate normal	shape: 0.0628 location: -0.0028 cov: 0.07385	[19]
From disease-free to progressed disease	Log-normal scale: 0.98 location: 1.40	shape (0.74, 1.22) location (1.21, 1.61)		Multivariate normal	scale: 0.1251 location: 0.0079 cov:0.07135	[19]
From progressed to death	0.04	(0.03, 0.05)		Beta (α, β)	0.53, 12.79	[20]
From metastasis to death	0.11	(0.10,0.120)		Beta (α, β)	2.38, 19.07	[20]
The proportion of patients receiving surgery in the metastasis state	0.42	Fix value		Beta (α, β)	27, 39	[19]
The proportion of patients receiving PET in the metastasis state	0.35	Fix value		Beta (α, β)	27, 42	[19]
**Utility**						
The stable state of surgery and PET						
Baseline value	0.75	(0.72, 0.79)		Beta (α, β)	7.13, 2.38	[21]
Progressed state of surgery and PET						
Decrements	-0.126	(-0.301, 0.049)		Normal (Mean, SE)	-0.126, 0.089	[22]
Metastatic state of surgery and PET						
Decrements	-0.352	(-0.493, -0.211)		Normal (Mean, SE)	-0.352, 0.072	[22]
Age decrement (annual)	-0.0013	-0.004, 0.002		Normal (Mean, SE)	-0.0013, 0.002	[23]
**Cost**						
Surgery	£6619.53					
Mastectomy, Unilateral Major Breast Procedures with Lymph Node Clearance, with CC Score 0–1 (JA38C)	£6547.64	Fixed value				[24], [25]
Proportion of mastectomy	35%	(28%-43%)		Beta (α, β)	16,340, 30,345	[13]
Breast-conserving surgery, Unilateral Major Breast Procedures with CC Score 0–2 (JA20F)	£2867.69	Fixed value				[24], [25]
Cost of hospital stays for surgery per cycle (WH20C)	£937.00	Fixed value				[13], [24], [25]
Cost of Tamoxifen (per tablet)	£0.30	Fixed value				[24], [25]
Cost of Letrozole (per tablet)	£3.25	Fixed value				[24], [25]
Cost of follow-up, Follow-up Examination for Malignant Neoplasm, without Interventions (WH52B)	£509.22	Fixed value				[24], [25]
Cost of progressed disease	£8251.75	(£5691.10-£11248.21)		Gamma (α, β)	33.88, 487.09	[26], [25]
Cost of metastatic disease	£6223.54	(£4990.12-£7638.91)		Gamma (α, β)	84.83, 146.73	[26], [25]

(Note) Values for the multivariate normal distribution obtained from Cholesky decomposition of the variance-covariance matrix, cov: covariance SE: standard error; CI: confident interval

### Clinical effectiveness

As effectiveness data for PET are relatively mature, a targeted literature review was performed to identify UK-based RCTs of PET with a long follow-up duration to reduce uncertainty in lifetime survival extrapolations. The effectiveness of PET was sourced from the RCT by Chakrabarti et al., which recruited participants (≥ 70 years) to receive tamoxifen alone and had a 20-year follow-up [19]. Kaplan-Meier curves for overall and progression-free survival were replicated using DigitizeIt^®^, and individual participant data were reconstructed according to Guyot et al. [27]. Parametric survival analysis (survival functions: Exponential, Weibull, Gompertz, Log-normal and Log-logistic) was performed [28] using Stata (Release 16.1, College Station, TX: StataCorp LLC). The most appropriate parametric survival function was selected based on the lowest Akaike Information Criteria (AIC) and Bayesian Information Criteria (BIC) values [29, 30], as well as visual inspection for clinical plausibility [28, 31] (Table 2). The effectiveness of surgery + ET was estimated by applying the hazard ratios for overall survival and progression-free survival (from the Cochrane review of surgery + ET versus PET) to the parametric survival curves for PET [10]. The proportion of individuals assumed to have metastatic disease within the progressed health state was estimated from the RCT by Chakrabarti et al. [19]. (reported in Supplementary Material 1 Appendix 2)

### Health utility

Health utility values were measured by the EQ-5D-3L instrument and valued by the UK tariff [30]. The EQ-5D-3L is a generic preference-based instrument for valuing health from 0 (dead) to 1 (perfect health) and states worse than dead are possible [31]. The EQ-5D-3L utility value for the stable health state was sourced from the RCT by Williams et al. [21]. Our model aggregated mastectomy and breast-conserving surgery (BCS) into a single ‘surgical intervention’ state, consistent with NICE guidelines (NG101) and evidence from Fteropoulli et al. [32] showing no significant long-term differences in quality-of-life outcomes between procedures beyond 12 months. Identical disutilities were applied, reflecting this convergence in recovery trajectories. Utility values for people experiencing progressed disease and metastasis were applied as linear decrements based on a systematic review and meta-regression of utility values for breast cancer (Table 2) [22]. To reflect the natural decline in quality of life as people age, an annual linear decrement of -0.001 was also applied, based on a meta-regression of EQ-5D utility values in older women with early-stage breast cancer [23]. QALYs were calculated by multiplying the duration of time in each health state by the corresponding health utility value.

### Resource use and cost

Resource use and cost were estimated to be representative of routine care within England. The UK’s national clinical guideline for early and locally advanced breast cancer was used to first describe the relevant pathways of care [8]. For surgical intervention, national audit data were used to estimate the proportion who received mastectomy, breast-conserving surgery and delayed breast reconstruction. It was assumed that (1) 35% of patients received mastectomy and 65% of patients received breast-conserving surgery [13]; (2) all patients receiving mastectomy would receive delayed breast reconstruction within one year [13]. The average unit cost of surgery was obtained from the NHS England National Cost Collection dataset (2019/2020) [24]. The cost of ET (for PET and adjuvant ET) was assumed that patients would receive tamoxifen in the first five years and then letrozole for an additional five years [8]. Long-term surgery-specific costs beyond initial treatment were excluded based on evidence that older surgical patients with clear margins experience recurrence rates (< 5% at 10 years) and mortality comparable to cancer-free peers [33], with no persistent surgery-attributable costs identified in costing studies [26]. The unit cost of treatments was obtained from the British National Formulary (BNF, November 2021, Drug Tariff). The costs of progressed and metastatic states were derived from a published costing study in England to reflect resource use and costs incurred in routine practice [26]. Historic prices were inflated to 2020/21 using the inflation indices by the Personal Social Services Research Unit [25]. Resource use and cost inputs were validated by clinicians.

### Data analysis

The results reported the incremental cost, life-years (LY) gained, QALYs gained, and incremental cost-effectiveness ratio (ICER). Cost-effectiveness was determined by comparing the estimated ICER with a threshold of £20,000 to £30,000 per QALY gained in accordance with the National Institute for Health and Care Excellence’s (NICE) reference case. The net monetary benefit (NMB) for each strategy and the incremental NMB (INMB) were calculated using cost-effectiveness thresholds of £20,000 and £30,000 per QALY gained [33, 34]. One-way sensitivity analysis of all input parameters was presented as a tornado diagram. A probabilistic sensitivity analysis (PSA) used Monte Carlo simulation (10,000 iterations) to handle uncertainty in all input parameters simultaneously. The results from the PSA were reported on a cost-effectiveness plane and cost-effectiveness acceptability curve (CEAC) to illustrate the probability of cost-effectiveness [35, 36].

### Value of implementation

A value of implementation analysis investigated the impact of improving the uptake of surgery + ET for older women with early-stage breast cancer [16]. The expected value of perfect implementation (EVPImp) per individual was estimated by subtracting the difference in the net benefit achieved from current levels of implementation for surgery + ET and PET from the net benefit achieved assuming perfect implementation of surgery + ET (cost-effectiveness threshold: £20,000 and £30,000 per QALY gained). The formula for estimating the EVPImp per individual is reported in Eq. 1, where $\:NB\left(j,\theta\:\right)$ refers to the net benefit of strategy $\:j$ (surgery + ET or PET) conditional on a set of $\:\theta\:$ uncertain input parameter values and $\:{\rho\:}_{j}\:$is the level of implementation for each comparator strategy where 0 ≤ r_j_ ≤ 1 and$\:\:\sum\:_{j=1}^{J}{\rho\:}_{j}=1$. The current proportions of individuals receiving PET (24%) and surgery + ET (76%) were estimated from the national audit of breast cancer in older people [13].

1 $$\:\begin{array}{c}EVPImp=\left[{\underset{\varvec{j}}{\varvec{m}\varvec{a}\varvec{x}}\varvec{E}}_{\varvec{\theta\:}}\varvec{N}\varvec{B}\left(\varvec{j},\varvec{\theta\:}\right)\right]-\left[\sum\:{\varvec{\rho\:}}_{\varvec{j}}{\varvec{E}}_{\varvec{\theta\:}}\varvec{N}\varvec{B}\left(\varvec{j},\varvec{\theta\:}\right)\right]\end{array}\:$$

Population-level EVPImp was estimated by scaling up these individual-level estimates by the future incident population. The size of this future incident population (*n* = 80,531) assumed an annual incidence of women aged 70 + years with ER + operable early-stage breast cancer from the national audit (*n* = 9,356), a 10-year time scale to estimate the cumulative population and a discount rate of 3.5% per year [13] (Population estimation process in Appendix 4). When expressed in QALYs, the population-level EVPImp represents the total health benefit forgone due to the imperfect implementation of surgery + ET. When expressed in monetary units, the population EVPImp represents the upper bound that the healthcare system should pay to support implementation strategies that improve the uptake of surgery + ET and remain cost-effective. The results were rounded to two decimal places.

## Result

### Base-case analysis

The results from the cost-effectiveness analysis are reported in Table 3. Estimated QALYs were higher for people receiving surgery + ET (4.57) than for PET (3.87). The lifetime incremental QALY gain for surgery + ET compared with PET was 0.71. Estimated costs were also higher for people receiving surgery + ET (£10,628) than for PET (£6,102). The lifetime incremental cost of surgery + ET was £4,526 compared with PET. The estimated ICER was £6,413 per QALY gained, which was below the cost-effectiveness threshold used by NICE. Therefore, surgery + ET was cost-effective compared with PET for older women with operable early-stage breast cancer.

**Table 3:** Results of base-case deterministic analysis

Results		Surgery + ET	PET
Expected cost (£)		£10,627.57	£6,101.76
Life year gained		15.75	13.94
Expected QALYs		4.57	3.87
Incremental cost (£)		£4,525.81	-
Incremental QALYs		0.71	-
ICER		£6,412.62 per QALY gain	-
Net monetary benefit (£)	λ = 20,000	£80,877.96	£71,288.45
	λ = 30,000	£126,630.72	£109,983.55
Incremental net monetary benefit (£)	λ = 20,000	£9,589.51	
	λ = 30,000	£16,647.17	

(Note): QALY: quality-adjusted life-years; ICER: incremental cost-effectiveness ratio

### Sensitivity analyses

**Fig. 2:**
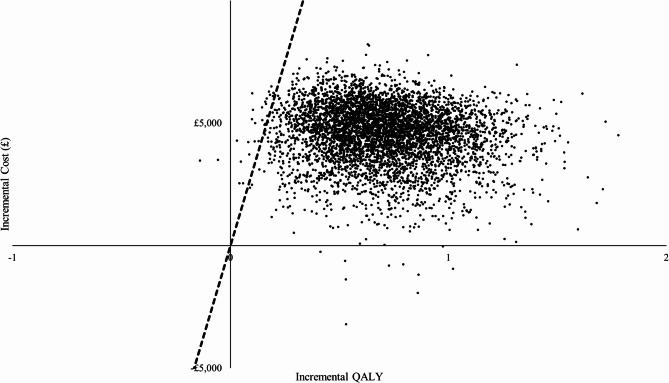
Cost-effectiveness plane of the probabilistic sensitivity analysis. (Note): Surgery + ET against PET, QALY: quality-adjusted life-year; CE: cost-effectiveness

**Fig. 3:**
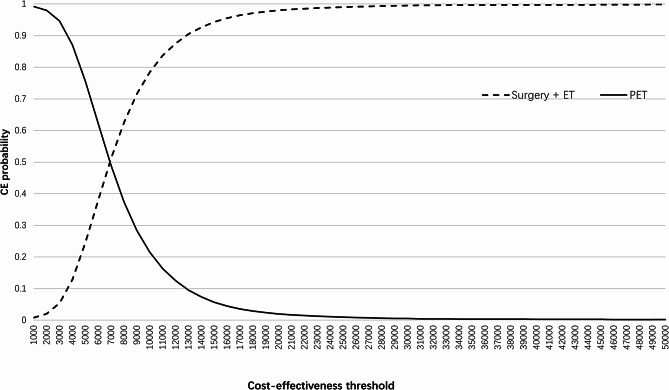
Cost-effectiveness acceptability curve. (Note) PET: Primary endocrine therapy

The deterministic one-way sensitivity analysis demonstrated that the base case result was robust to uncertainty in all input parameter values. The incremental net monetary benefit of surgery + ET remained positive for all input parameter sensitivity analyses. As visualised in the tornado diagram (Appendix 3), the overall survival and progression-free survival of PET and surgery are the key drivers of the estimated ICER’s magnitude. However, in all cases, surgery + ET remained cost-effective compared with PET. Figure 2 reports that most points from the probabilistic sensitivity analysis fell within the north-east quadrant of the cost-effectiveness plane, indicating that surgery + ET is highly likely to increase health outcomes and costs. The probabilistic mean costs of surgery + ET and PET were £10,682.54 and £6,122.27, and the mean QALYs of surgery + ET and PET were 4.58 and 3.87, respectively. The probabilistic mean life-year gained of surgery + ET and PET were 15.81 and 13.98, respectively. The CEAC in Fig. 3 reports that surgery + ET had a 98% probability of being cost-effective at a threshold of £20,000 per QALY gained and a 99.5% probability of being cost-effective at a threshold of £30,000 per QALY gained.

### Value of implementation analysis

At the threshold of £20,000 and £30,000, the estimated net benefit per individual under the perfect implementation of surgery + ET was 4.04 QALYs or £80,877.96, and 4.22 QALYs or £126,630.72, respectively. The estimated net benefit per individual based on current levels of implementation was 3.93 QALYs or £78,576.48, and 4.09 QALYs or £122,635.40, respectively. Therefore, the EVPImp per individual was 0.12 QALYs or £2301.48, and 0.13 QALYs or £3,995.32, respectively. At the population level, at the threshold of £20,000 and £30,000, the EVPImp expressed in health outcomes was 9,267 and 10,725 QALYs, respectively, representing the current disease burden and benefits forgone due to the imperfect implementation of surgery + ET. The population-level EVPImp expressed in monetary units was £185,340,619.79 (£0.19 billion) and £321,747,086.46 (£0.32 billion), respectively. The results indicate that implementation strategies to improve the uptake of surgery + ET are likely to be cost-effective.

## Discussion

This study aimed to estimate the cost-effectiveness and value of implementation of surgery plus ET compared with PET for older women with early breast cancer who are fit for surgery. The findings confirm that surgical intervention with adjuvant ET is cost-effective compared with PET for this population. These findings align with current clinical guidelines while highlighting substantial health economic implications: the observed trend toward PET utilisation resulted in 9,267 QALYs forgone at current implementation levels at the threshold of £20,000. Rather than advocating for reduced PET use, we emphasise that within shared decision-making frameworks, clinicians should actively consider both the clinical and economic benefits of Surgery + ET when clinically appropriate. We therefore recommend integrating these economic insights into patient-clinician discussions to support value-informed choices, particularly for patients where surgical feasibility aligns with personal treatment goals.

There is a growing need for evidence supporting ways to improve the cost-effectiveness of healthcare for older people diagnosed with early-stage breast cancer. The most relevant previous study related to the present findings was by Holmes et al., who reported a model-based health economic evaluation of surgery with adjuvant ET compared with PET for older women [12]. Unlike the present findings, Holmes et al. estimated that surgery + ET was associated with lower costs than PET [12]. One possible explanation for this difference was that, following the recommendations within the NICE clinical guideline for breast cancer [8], the present study included the cost of breast reconstruction for all individuals who received a mastectomy. Irrespective of this difference in the estimated incremental cost between the two studies, the magnitude of health gain associated with surgery + ET was large enough to offset the higher cost of surgical intervention. In addition, for the present study, surgery was defined as either mastectomy or BCS. This decision was appropriate because these surgical strategies are non-inferior to each other in terms of 10-year overall survival when combined with an appropriate adjuvant therapy [37, 38]. As a result, the interpretation of the findings aligns with those of Holmes et al.; both studies show that surgery + ET is cost-effective for older women who are fit for surgery [12].

There are several reasons why older people with early-stage breast cancer may receive PET instead of surgery within routine clinical practice. Treatment decisions for breast cancer are made within a shared decision-making environment, which is shaped by the preferences of those receiving and providing care. However, the findings from this study show that widespread use of PET amongst individuals who are eligible for surgery corresponds with lower health outcomes at the individual and population levels. The results from this study may help support future shared decision-making consultations by providing evidence on quality-of-life outcomes and life expectancy. Meanwhile, multidisciplinary insights, including input from a UK breast surgeon and a clinician survey [39], confirmed that non-surgical management in older women is primarily driven by frailty, comorbidities, cognitive barriers, and quality-of-life preferences, consistent with national MDT decision-making. Moreover, the findings provide healthcare decision-makers with evidence to justify exploring population-level implementation strategies to improve the uptake of first-line surgical interventions.

PET may have a more promising role in managing older people with early-stage breast cancer who are not fit for surgery. For example, surgery is likely to pose higher risks for those individuals with significant comorbidities or high levels of frailty. Understanding the effectiveness and cost-effectiveness of PET within this subpopulation will be a valuable topic for future research to help support management decisions for these individuals who experience a high burden of disease relative to those eligible for surgery.

While the EVPImp quantifies potential health gains, achieving perfect implementation would require investments in patient education, clinician training, and systemic support. These costs were not modeled here but could offset net benefits. When expressed in monetary units, the EVPImp represents the upper-bound on the cost of any implementation activities to be cost-effective for the healthcare system. Future research should evaluate the cost-effectiveness of alternative implementation strategies (e.g., decision aids, audit-feedback) within an expected value of specific implementation framework to prioritise implementation strategies based on their expected cost and likely benefit [16].

The present study has several key strengths. First, the value of implementation analysis provides a unique contribution to the debate about using PET for older people with early-stage breast cancer. The findings will give healthcare decision-makers much-needed evidence about the potential consequences of using PET in routine practice. Second, using RCT data with a 20-year follow-up period meant that parameter uncertainty within lifetime survival extrapolations was reduced substantially.

However, there are limitations to this study. First, the underlying RCT data did not report results by ER status. The underlying RCT was selected from a meta-analysis [11], although there were also other RCTs that recruited patients with selective ER positivity. Our team performed a sensitivity analysis using the RCT by Johnston and colleagues, which included older women with ER + operable primary breast cancer with a 20-year follow-up [40], confirming that surgery + ET remains a cost-effective strategy, which aligns with the cost-effectiveness by Holmes [12] (Appendix 5). The Johnston et al. trial, which included clearer ER + subgroup analyses, consistently reinforced our core finding that surgery remains cost-effective for older women clinically suitable for operative intervention. Therefore, although the effectiveness of PET may be underestimated if the sample included individuals who were less likely to benefit because they were not ER+, these analyses indicate the upper bound of the benefit of surgery + ET [41, 42]. To assess the external validity of the model-based analysis, the estimated overall survival was compared with outcomes observed in routine care within England. A recent analysis of linked Clinical Practice Research Datalink (CPRD), Hospital Episode Statistics, National Cancer Registry, and Death Registration demonstrated that for people with breast cancer who were at least 70 years old with low comorbidity and no frailty, the median overall survival for surgery + ET was 6.8 years (IQR: 4.0–10.6), and for PET, it was 3.2 years (IQR: 1.4–6.0). In comparison, the model-based analysis in the present study estimated a median overall survival of 6.0–6.5 years for surgery + ET and 5.5–6.0 years for PET [43]. This comparison illustrated that the model-based analysis simulated overall survival outcomes that were similar to the outcomes observed for routine care within an independent dataset for England, and enhances the credibility of the findings.

Second, the estimated incident population for the value of implementation analysis assumed that all individuals who did not receive surgical intervention received PET instead. In practice, this may have overestimated the proportion receiving PET, and the value of implementation analysis results should be interpreted as an upper bound. Third, while RCT data reduce uncertainty in survival extrapolation, they may not fully reflect real-world effectiveness due to strict inclusion criteria and standardised protocols. For instance, older patients in routine practice often have higher comorbidity burdens than RCT participants, potentially overestimating the benefits of surgery + ET. Finally, the value of implementation analysis assumed all non-surgical patients received PET. In practice, some patients who are eligible for surgery may receive a different non-surgical intervention such as targeted therapy or radiotherapy. By assuming that the comparator arm reflected PET only rather than a weighted average of alternative treatments, the value of implementing surgery plus adjutant ET may be overestimated reinforcing that these findings should be interpreted as an upper-bound.

Third, while QALYs are the preferred outcome measure for health economic analyses to support decision-making within the UK, they may not fully capture clinical outcomes such as disease-free survival or patient-reported quality of life measured by instruments like the EORTC QLQ-30. Policymakers in other healthcare jurisdictions and clinicians may find QALYs challenging to interpret compared to direct clinical endpoints. Future studies could strengthen the analysis by incorporating disease-free survival rates or validated patient-reported outcome measures to complement QALY estimates. Finally, the model’s three health states may not fully reflect the complexity and heterogeneity of individual clinical pathways, such as the specific quality-of-life impact of mastectomy in elderly women or the cumulative burden of sequential salvage therapies following multiple progressions, as these are aggregated within the broader progression-free and progressed states.

Future research could undertake a value of implementation analysis comparing surgery with adjuvant ET and PET within different healthcare jurisdictions by using data on the uptake of these interventions for older people with early-stage breast cancer in different countries. Future observational data analysis will also be valuable in estimating the relative effectiveness of PET for people who cannot receive surgical interventions due to frailty or comorbidities [44]. Second, to support healthcare decision-making, future model-based economic evaluations of PET will be essential to investigate the cost-effectiveness of non-surgical intervention amongst these subpopulations of individuals experiencing frailty or comorbidities. Shifting practice toward guideline-recommended care necessitates addressing patient and clinician preferences. For example, addressing surgical apprehensions through shared decision-making tools or reducing frailty-related risks through preoperative optimisation may require dedicated funding. The cost-effectiveness of these provider-facing implementation strategies should be evaluated by future research to ensure net benefits are sustained. Finally, this analysis adopted a healthcare system perspective, excluding societal costs such as caregiver burden or productivity losses. A societal perspective might reveal additional economic impacts, particularly for older populations reliant on informal care. Future evaluations could explore this dimension.

## Conclusion

Surgery with adjuvant ET is highly likely to be more effective and cost-effective than PET for older women with operable early-stage breast cancer. Healthcare decision-makers and care providers can achieve substantial population health benefits by reducing PET within this population. Nonetheless, PET may hold promise as a cost-effective alternative for frail older women with early-stage breast cancer, which should become the focus of future health economic research.

## Supplementary Information

Below is the link to the electronic supplementary material.

Supplementary Material 1

Supplementary Material 2

## Data Availability

No datasets were generated or analysed during the current study.

## References

[1] Allemani C, et al. Global surveillance of trends in cancer survival 2000–14 (CONCORD-3): analysis of individual records for 37 513 025 patients diagnosed with one of 18 cancers from 322 population-based registries in 71 countries. Lancet. 2018;391(10125):1023–75. 29395269 10.1016/S0140-6736(17)33326-3 PMC5879496

[2] Cancer Research UK. Breast cancer survival statistics: Breast cancer survival trend over time, 1971–2011. 2014 [cited 2020 28 October]; Available from: https://www.cancerresearchuk.org/health-professional/cancer-statistics/statistics-by-cancer-type/breast-cancer/survival?utm_source=affiliate_window&utm_medium=affiliate&utm_name=online_retail&utm_content=www.flexoffers.com&awc=2584_1603922913_237f8c0580cfa4ad9568c842fc66ae96#heading-Two

[3] Hu K et al. Global patterns and trends in the breast cancer incidence and mortality according to sociodemographic indices: an observational study based on the global burden of diseases. 2019;9(10):e028461. 10.1136/bmjopen-2018-028461 PMC6797270 31594871

[4] Laudicella M, et al. Cost of care for cancer patients in england: evidence from population-based patient-level data. Br J Cancer. 2016;114(11):1286–92. 27070711 10.1038/bjc.2016.77 PMC4891510

[5] Wittenberg R et al. Care for older people: projected expenditure to 2022 on social care and continuing health care for England’s older population. 2012 [cited 2020 24 March]; Available from: https://www.nuffieldtrust.org.uk/research/care-for-older-people-projected-expenditure-to-2022-on-social-care-and-continuing-health-care-for-england-s-older-population#partners

[6] Dehal A, Abbas A, Johna S. Comorbidity and outcomes after surgery among women with breast cancer: analysis of nationwide in-patient sample database. Breast Cancer Res Treat. 2013;139(2):469–76. 23624816 10.1007/s10549-013-2543-9

[7] Anim-Sampong A, et al. Psychosocial impact of mastectomy on female breast cancer patients presenting at an academic radiotherapy oncology centre in Ghana. I Radiother Pract. 2021;20(3):306–15.

[8] National Institute for Health and Care Excellence. Early and locally advanced breast cancer: diagnosis and management. 2018 [cited 2018 Jul]; Available from: https://www.nice.org.uk/guidance/ng101/evidence 40601805

[9] Biganzoli L, et al. The requirements of a specialist breast centre. Breast. 2020;51:65–84. 32217457 10.1016/j.breast.2020.02.003 PMC7375681

[10] Morgan J, et al. Surgery versus primary endocrine therapy for operable primary breast cancer in elderly women (70 years plus). Cochrane Database Syst Rev. 2014;5:CD004272. 35658165 10.1002/14651858.CD004272.pub3 PMC9645779

[11] Chan KS, et al. Revisiting primary endocrine therapy versus surgery in older women with breast cancer: meta-analysis. Br J Surg. 2023;110(4):420–31. 36718056 10.1093/bjs/znac435

[12] Holmes GR, et al. Cost-Effectiveness modeling of surgery plus adjuvant endocrine therapy versus primary endocrine therapy alone in UK women aged 70 and over with early breast cancer. Value Health. 2021;24(6):770–9. 34119074 10.1016/j.jval.2020.12.016

[13] Gannon M et al. National Audit of Breast Cancer in Older Patients: 2022 Annual Report. The Royal College of Surgeons 2022;94.

[14] Burton M, et al. The information and decision support needs of older women (> 75 yrs) facing treatment choices for breast cancer: a qualitative study. Psychooncology. 2015;24(8):878–84. 25534045 10.1002/pon.3735

[15] Wylie S, Ravichandran D. A UK National survey of breast surgeons on primary endocrine therapy of early operable breast cancer. Ann R Coll Surg Engl. 2013;95(5):353–6. 23838499 10.1308/003588413X13629960045832 PMC4165139

[16] Fenwick E, Claxton K, Sculpher M. The value of implementation and the value of information: combined and uneven development. Med Decis Mak. 2008;28(1):21–32. 10.1177/0272989X07308751 18263559

[17] Husereau D, et al. Consolidated health economic evaluation reporting standards 2022 (CHEERS 2022) statement: updated reporting guidance for health economic evaluations. Value Health. 2022;25(1):3–9. 35031096 10.1016/j.jval.2021.11.1351

[18] Wang Y, et al. Systematic review of the evidence sources applied to cost-effectiveness analyses for older women with primary breast cancer. Cost Eff Resource Allocation. 2022;20(1):9. 10.1186/s12962-022-00342-7 PMC8889747 35232445

[19] Chakrabarti J, et al. A randomised trial of mastectomy only versus Tamoxifen for treating elderly patients with operable primary breast cancer-final results at 20-year follow-up. Crit Rev Oncol Hematol. 2011;78(3):260–4. 20447833 10.1016/j.critrevonc.2010.04.006

[20] Erman A, et al. Cost-effectiveness analysis of extended adjuvant endocrine therapy in the treatment of post-menopausal women with hormone receptor positive breast cancer. Breast Cancer Res Treat. 2014;145(2):267–79. 24771048 10.1007/s10549-014-2950-6

[21] Williams LJ, et al. A randomised controlled trial of post-operative radiotherapy following breast-conserving surgery in a minimum-risk population. Quality of life at 5 years in the PRIME trial. Health Technol Assess. 2011;15(12):i–xi. 21366974 10.3310/hta15120

[22] Peasgood T, Ward SE, Brazier J. Health-state utility values in breast cancer. Expert Rev Pharmacoecon Outcomes Res. 2010;10(5):553–66. 20950071 10.1586/erp.10.65

[23] Wang Y, et al. The impact of age on health utility values for older women with early-stage breast cancer: a systematic review and meta-regression. Health Qual Life Outcomes. 2022;20(1):169. 36564800 10.1186/s12955-022-02067-w PMC9789668

[24] National Health Service. 2019/20 National Cost Collection data Version 2. 2019–2020 [cited 2021 27 Dec]; Available from: https://www.england.nhs.uk/national-cost-collection/

[25] Personal Social Services Research Unit. Unit costs of health and social care 2021. University of Kent; 2021. p. 29.

[26] Karnon J, et al. Health care costs for the treatment of breast cancer recurrent events: estimates from a UK-based patient-level analysis. Br J Cancer. 2007;97(4):479–85. 17653077 10.1038/sj.bjc.6603887 PMC2360350

[27] Guyot P, et al. Enhanced secondary analysis of survival data: reconstructing the data from published Kaplan-Meier survival curves. BMC Med Res Methodol. 2012;12(1):9. 22297116 10.1186/1471-2288-12-9 PMC3313891

[28] Ishak KJ, et al. Overview of parametric survival analysis for health-economic applications. PharmacoEconomics. 2013;31(8):663–75. 23673905 10.1007/s40273-013-0064-3

[29] Latimer N. NICE DSU technical support document 14: survival analysis for economic evaluations alongside clinical trials-extrapolation with patient-level data. Report by the Decision Support Unit; 2011. 27905716

[30] Akaike H. A new look at the statistical model identification. IEEE Trans Autom Control. 1974;19(6):716–23.

[31] Connock M, Hyde C, Moore D. Cautions regarding the fitting and interpretation of survival curves. PharmacoEconomics. 2011;29(10):827–37. 21770482 10.2165/11585940-000000000-00000

[32] Fteropoulli T, et al. Changes in health-related quality of life following breast cancer surgery: A systematic review of the literature on the role of surgical approaches. Eur J Surg Oncol. 2025;51(1):109467. 39580262 10.1016/j.ejso.2024.109467

[33] Hughes KS, et al. Lumpectomy plus Tamoxifen with or without irradiation in women age 70 years or older with early breast cancer: long-term follow-up of CALGB 9343. J Clin Oncol. 2013;31(19):2382–7. 23690420 10.1200/JCO.2012.45.2615 PMC3691356

[33] York Health Economics Consortium. Net Monetary Benefit. 2016 [cited 2022 25 April]; Available from: https://yhec.co.uk/glossary/net-monetary-benefit/

[34] Gandjour A. Willingness to pay for new medicines: a step towards narrowing the gap between NICE and IQWiG. BMC Health Serv Res. 2020;20(1):343. 32321496 10.1186/s12913-020-5050-9 PMC7178559

[35] O’Hagan A, Stevenson M, Madan J. Monte Carlo probabilistic sensitivity analysis for patient level simulation models: efficient Estimation of mean and variance using ANOVA. Health Econ. 2007;16(10):1009–23. 17173339 10.1002/hec.1199

[36] Fenwick E, Claxton K, Sculpher M. Representing uncertainty: the role of cost-effectiveness acceptability curves. Health Econ. 2001;10(8):779–87. 11747057 10.1002/hec.635

[37] Rajan KK et al. Overall survival after mastectomy versus breast-conserving surgery with adjuvant radiotherapy for early-stage breast cancer: meta-analysis. BJS Open, 2024;8(3). 10.1093/bjsopen/zrae040 PMC11100524 38758563

[38] Shrestha P, et al. Higher 10-Year survival with breast-Conserving therapy over mastectomy for women with Early-Stage (I-II) breast cancer: analysis of the CDC patterns of care data base. Breast Cancer (Auckl). 2024;18:11782234241273666. 39328281 10.1177/11782234241273666 PMC11425729

[39] Morgan JL, et al. Healthcare professionals’ preferences for surgery or primary endocrine therapy to treat older women with operable breast cancer. Eur J Surg Oncol. 2015;41(9):1234–42. 26108734 10.1016/j.ejso.2015.05.022

[40] Johnston SJ, et al. A randomised trial of primary Tamoxifen versus mastectomy plus adjuvant Tamoxifen in fit elderly women with invasive breast carcinoma of high oestrogen receptor content: long-term results at 20 years of follow-up. Ann Oncol. 2012;23(9):2296–300. 22357257 10.1093/annonc/mdr630

[41] Fujii T, et al. Revisiting the definition of Estrogen receptor positivity in HER2-negative primary breast cancer. Ann Oncol. 2017;28(10):2420–8. 28961844 10.1093/annonc/mdx397 PMC5834134

[42] Paakkola NM, et al. The prognostic and predictive impact of low Estrogen receptor expression in early breast cancer: a systematic review and meta-analysis. ESMO Open. 2021;6(6):100289. 34678571 10.1016/j.esmoop.2021.100289 PMC8531568

[43] Wang Y, et al. Survival outcomes in older women with Oestrogen-Receptor-Positive Early-Stage breast cancer: primary endocrine therapy vs. Surgery by comorbidity and frailty levels. Cancers. 2024;16. 10.3390/cancers16040749 10.3390/cancers16040749 PMC10886896 38398140

[44] Berger ML, et al. Good practices for real-world data studies of treatment and/or comparative effectiveness: recommendations from the joint ISPOR-ISPE special task force on real-world evidence in health care decision making. Pharmacoepidemiol Drug Saf. 2017;26(9):1033–9. 28913966 10.1002/pds.4297 PMC5639372

