# Supplementary material for: Primary endocrine therapy versus surgery plus endocrine therapy for early-stage breast cancer in older women without frailty: a cost-effectiveness and value of implementation analysis
Source: Health Econ Rev. 2025 Sep 30;15:75. doi: 10.1186/s13561-025-00668-x (PMC12487327; doi:10.1186/s13561-025-00668-x)
Supplement: Supplementary file 1 — Supplementary Material 1 [file 13561_2025_668_MOESM1_ESM.docx]

**Primary Endocrine Therapy Versus Surgery Plus Endocrine Therapy for Early-Stage Breast Cancer in Older Women without Frailty: A Cost-effectiveness and Value of Implementation Analysis**

**Supplementary Appendix**

[Appendix 1. CHEERS 2022 Checklist 2](#_Toc174005844)

[Appendix 2. Estimating time to death and progression 4](#_Toc174005845)

[Appendix 3 One-way Sensitivity Analyses 7](#_Toc174005846)

[Appendix 4 Population Estimation for the Value of Implementation Analysis 8](#_Toc174005847)

[Appendix 5. Sensitivity analysis using RCT by Johnston 9](#_Toc174005848)

# Appendix 1. CHEERS 2022 Checklist

| **Topic** | **No.** | **Item** | **Location where item is reported** |
| --- | --- | --- | --- |
| **Title** |  |  |  |
|  | 1 | Identify the study as an economic evaluation and specify the interventions being compared. | Title, Page 1 |
| **Abstract** |  |  |  |
|  | 2 | Provide a structured summary that highlights context, key methods, results, and alternative analyses. | Abstract, Page 2 |
| **Introduction** |  |  |  |
| **Background and objectives** | 3 | Give the context for the study, the study question, and its practical relevance for decision making in policy or practice. | Introduction |
| **Methods** |  |  |  |
| **Health economic analysis plan** | 4 | Indicate whether a health economic analysis plan was developed and where available. | Methods, Study design & Table 1 |
| **Study population** | 5 | Describe characteristics of the study population (such as age range, demographics, socioeconomic, or clinical characteristics). | Methods, Study design |
| **Setting and location** | 6 | Provide relevant contextual information that may influence findings. | Methods, Study design |
| **Comparators** | 7 | Describe the interventions or strategies being compared and why chosen. | Methods, Study design |
| **Perspective** | 8 | State the perspective(s) adopted by the study and why chosen. | Methods, Model structure |
| **Time horizon** | 9 | State the time horizon for the study and why appropriate. | Methods, Model structure |
| **Discount rate** | 10 | Report the discount rate(s) and reason chosen. | Methods, Study design |
| **Selection of outcomes** | 11 | Describe what outcomes were used as the measure(s) of benefit(s) and harm(s). | Methods, Clinical Effectiveness, Health Utility |
| **Measurement of outcomes** | 12 | Describe how outcomes used to capture benefit(s) and harm(s) were measured. | Methods, Clinical Effectiveness, Health Utility |
| **Valuation of outcomes** | 13 | Describe the population and methods used to measure and value outcomes. | Methods, Health Utility |
| **Measurement and valuation of resources and costs** | 14 | Describe how costs were valued. | Methods, Resource use and cost |
| **Currency, price date, and conversion** | 15 | Report the dates of the estimated resource quantities and unit costs, plus the currency and year of conversion. | Methods, Resource use and cost |
| **Rationale and description of model** | 16 | If modelling is used, describe in detail and why used. Report if the model is publicly available and where it can be accessed. | Methods，Model structure |
| **Analytics and assumptions** | 17 | Describe any methods for analysing or statistically transforming data, any extrapolation methods, and approaches for validating any model used. | Methods, Data analysis |
| **Characterising heterogeneity** | 18 | Describe any methods used for estimating how the results of the study vary for subgroups. | Methods, Data analysis |
| **Characterising distributional effects** | 19 | Describe how impacts are distributed across different individuals or adjustments made to reflect priority populations. | Methods, Data analysis |
| **Characterising uncertainty** | 20 | Describe methods to characterise any sources of uncertainty in the analysis. | Methods Data analysis |
| **Approach to engagement with patients and others affected by the study** | 21 | Describe any approaches to engage patients or service recipients, the general public, communities, or stakeholders (such as clinicians or payers) in the design of the study. | Not reported |
| **Results** |  |  |  |
| **Study parameters** | 22 | Report all analytic inputs (such as values, ranges, references) including uncertainty or distributional assumptions. | Methods Table 2 |
| **Summary of main results** | 23 | Report the mean values for the main categories of costs and outcomes of interest and summarise them in the most appropriate overall measure. | Results, Base-case analysis |
| **Effect of uncertainty** | 24 | Describe how uncertainty about analytic judgments, inputs, or projections affect findings. Report the effect of choice of discount rate and time horizon, if applicable. | Results, Sensitivity analyses |
| **Effect of engagement with patients and others affected by the study** | 25 | Report on any difference patient/service recipient, general public, community, or stakeholder involvement made to the approach or findings of the study | Not available |
| **Discussion** |  |  |  |
| **Study findings, limitations, generalisability, and current knowledge** | 26 | Report key findings, limitations, ethical or equity considerations not captured, and how these could affect patients, policy, or practice. | Discussion |
| **Other relevant information** |  |  |  |
| **Source of funding** | 27 | Describe how the study was funded and any role of the funder in the identification, design, conduct, and reporting of the analysis | End of manuscript |
| **Conflicts of interest** | 28 | Report authors conflicts of interest according to journal or International Committee of Medical Journal Editors requirements. | End of manuscript |

*From:* Husereau D, Drummond M, Augustovski F, et al. Consolidated Health Economic Evaluation Reporting Standards 2022 (CHEERS 2022) Explanation and Elaboration: A Report of the ISPOR CHEERS II Good Practices Task Force. Value Health 2022;25. <doi:10.1016/j.jval.2021.10.008>

# Appendix 2. Estimating time to death and progression

Parametric survival analysis was used to extrapolate the overall survival for PET, assuming the risk of death change over time and estimating the survival functions and probability functions at a particular time point [1, 2]. A randomised control trial was used to identify the survival probabilities for older women with primary breast cancer receiving surgery and primary endocrine therapy with 20 years of observation since the trial started in the early 1980s [3].

The five parametric survival curves described (exponential, Weibull, Gompertz, log-logistic, log-normal) fit these survival data. The parametric survival curve used to simulate time to death in the decision-analytic model was chosen according to the lowest AIC and BIC statistics and by visual inspection to ensure the biological plausibility of the estimated survival curve. All analyses were performed in Stata Version 14.

**Results**

The Kaplan-Meier curves of initial surgery and PET for all-cause mortality and time to progression were plotted separately.


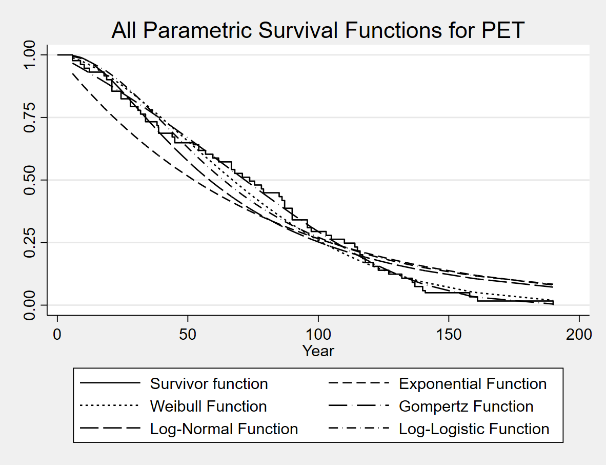
Kaplan-Meier curves of surgery and PET for all-cause mortality

Proportion Surviving

(Note) The left figure is the curve plotted for the initial surgery, and the right figure is the curve for the PET.

Parametric survival distribution of PET for all-cause mortality

| Parameter | Exponential | Weibull | Gompertz | Log-normal | Log-logistic |
| --- | --- | --- | --- | --- | --- |
| Rate | -1.94 |  |  |  |  |
| Shape |  | -3.19 | -2.38 | 1.7 | 1.72 |
| Scale |  | 0.45 | 0.082 |  | 0.41 |
| Location |  |  |  | 0.73 |  |
| AIC | 254.20 | 230.08 | 244.19 | 227.79 | 226.52 |
| BIC | 256.80 | 235.29 | 249.40 | 233.0 | 231.73 |

The Log-logistic survival curves had the lowest AIC and BIC of PET, indicating that it best fits the observed data. Therefore, the decision-analytic model used a Log-logistic survival curve of PET to estimate the time to death.

Kaplan-Meier curves of surgery and PET for time to progression


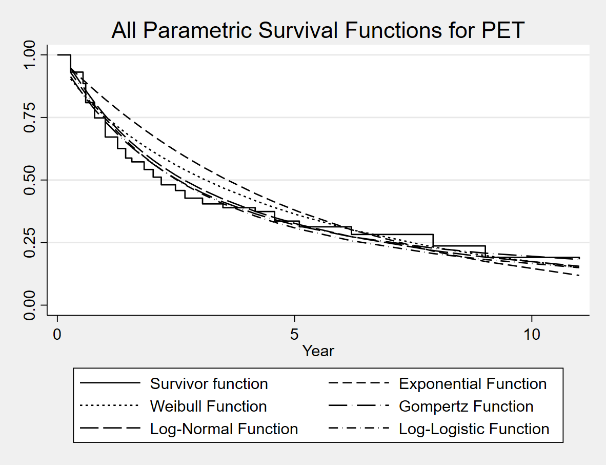


Parametric survival distribution of PET for time to progression

| Parameter | Exponential | Weibull | Gompertz | Log-normal | Log-logistic |
| --- | --- | --- | --- | --- | --- |
| Rate | -2.11 |  |  |  |  |
| Shape |  | -1.22 | -0.57 | 1.39 | 1.26 |
| Scale |  | -0.61 | -0.62 |  | 1.3 |
| Location |  |  |  | 0.98 |  |
| AIC | 460.10 | 453.29 | 440.34 | 431.15 | 436.52 |
| BIC | 462.97 | 459.04 | 446.09 | 436.90 | 442.27 |

The Log-normal survival curves had the lowest AIC and BIC test statistics of PET, indicating that it fits the observed data best. Therefore, the decision-analytic model used a Log-normal survival curve of PET to estimate the time to progression.

# Appendix 3 One-way Sensitivity Analyses

The one-way sensitivity analyses reported in the Tornado diagram (**Figure 1**) indicated that the INMB is most sensitive to the parameter uncertainty associated with the transition probabilities from the stable to dead states. The positive results of INMB for all the parameters suggested that decision-making regarding PET not being cost-effective will not change despite existing uncertainty. The results are robust to changes in the input parameter values.

Figure 1. Tornado diagram for one-way sensitivity analysis

# Appendix 4 Population Estimation for the Value of Implementation Analysis

The analysis focused on older women aged 70 years and above with ER+ operable early-stage breast cancer. The population size for the Value of Implementation Analysis was estimated using data from the 2022 National Audit of Breast Cancer in Older Women [4]. According to the audit, the annual incidence of newly diagnosed breast cancer in the UK was 55,000 cases. Among these, 38% were patients aged over 70, with 76% scoring between 0 and 1 on the Charlson Comorbidity Index. Of the older women, 58.9% were ER-positive. Consequently, there were 9,356 older women aged 70 years and above with ER+ operable early-stage breast cancer per year. When scaling this incidence to a 10-year horizon with a discount rate of 3.5%, the figure amounted to 80,531.

# Appendix 5. Sensitivity analysis using RCT by Johnston

Parametric survival analysis was used to extrapolate the overall survival for PET, assuming the risk of death change over time and estimating the survival functions and probability functions at a particular time point [1, 2]. A randomised control trial by Johnston was used to identify the survival probabilities for older women with primary breast cancer receiving surgery and primary endocrine therapy with 20 years of observation since the trial started in the early 1980s [5].

The five parametric survival curves described (exponential, Weibull, Gompertz, log-logistic, log-normal) fit these survival data. The parametric survival curve used to simulate time to death in the decision-analytic model was chosen according to the lowest AIC and BIC statistics and by visual inspection to ensure the biological plausibility of the estimated survival curve. All analyses were performed in Stata Version 14.

**Results**

The Kaplan-Meier curves of initial surgery and PET for all-cause mortality and time to progression were plotted separately.

Kaplan-Meier curves of surgery and PET for all-cause mortality


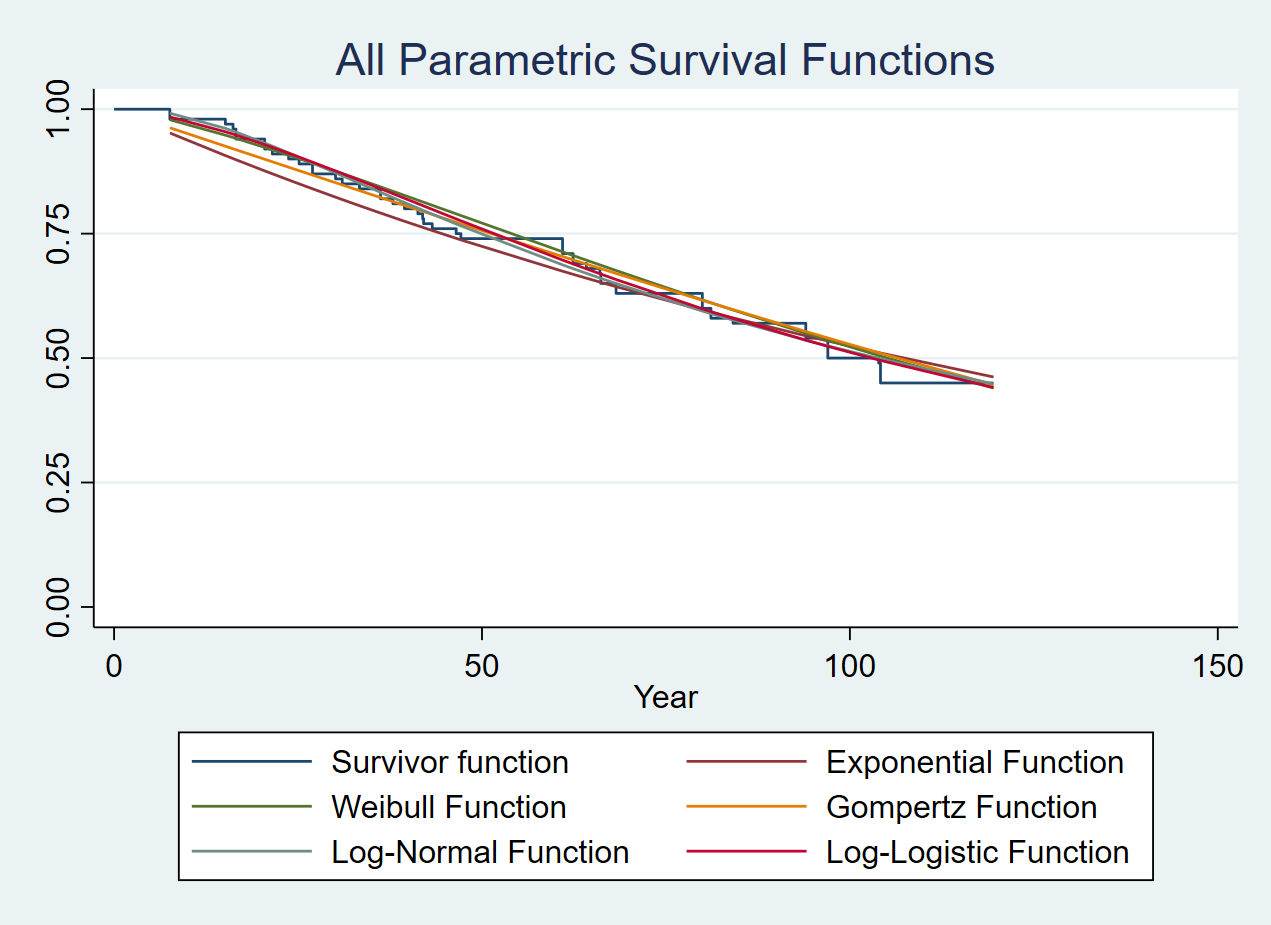


Proportion Surviving

Time (Month)

(Note) The left figure is the curve plotted for the initial surgery, and the right figure is the curve for the PET.

Parametric survival distribution of PET for all-cause mortality

| Parameter | Exponential | Weibull | Gompertz | Log-normal | Log-logistic |
| --- | --- | --- | --- | --- | --- |
| Rate | -5.04 |  |  |  |  |
| Shape |  | -6.51 | -5.32 | 4.64 | 4.63 |
| Scale |  | 1.32 | 0.005 |  | 0.63 |
| Location |  |  |  | 1.09 |  |
| AIC | 242.54 | 239.87 | 242.91 | 236.49 | 237.93 |
| BIC | 245.14 | 245.09 | 248.12 | 241.70 | 243.14 |

The Log-normal survival curves had PET's lowest AIC and BIC, indicating that it best fits the observed data. Therefore, the decision-analytic model used a Logistic survival curve of PET to estimate the time to death.

Kaplan-Meier curves of surgery and PET for time to progression


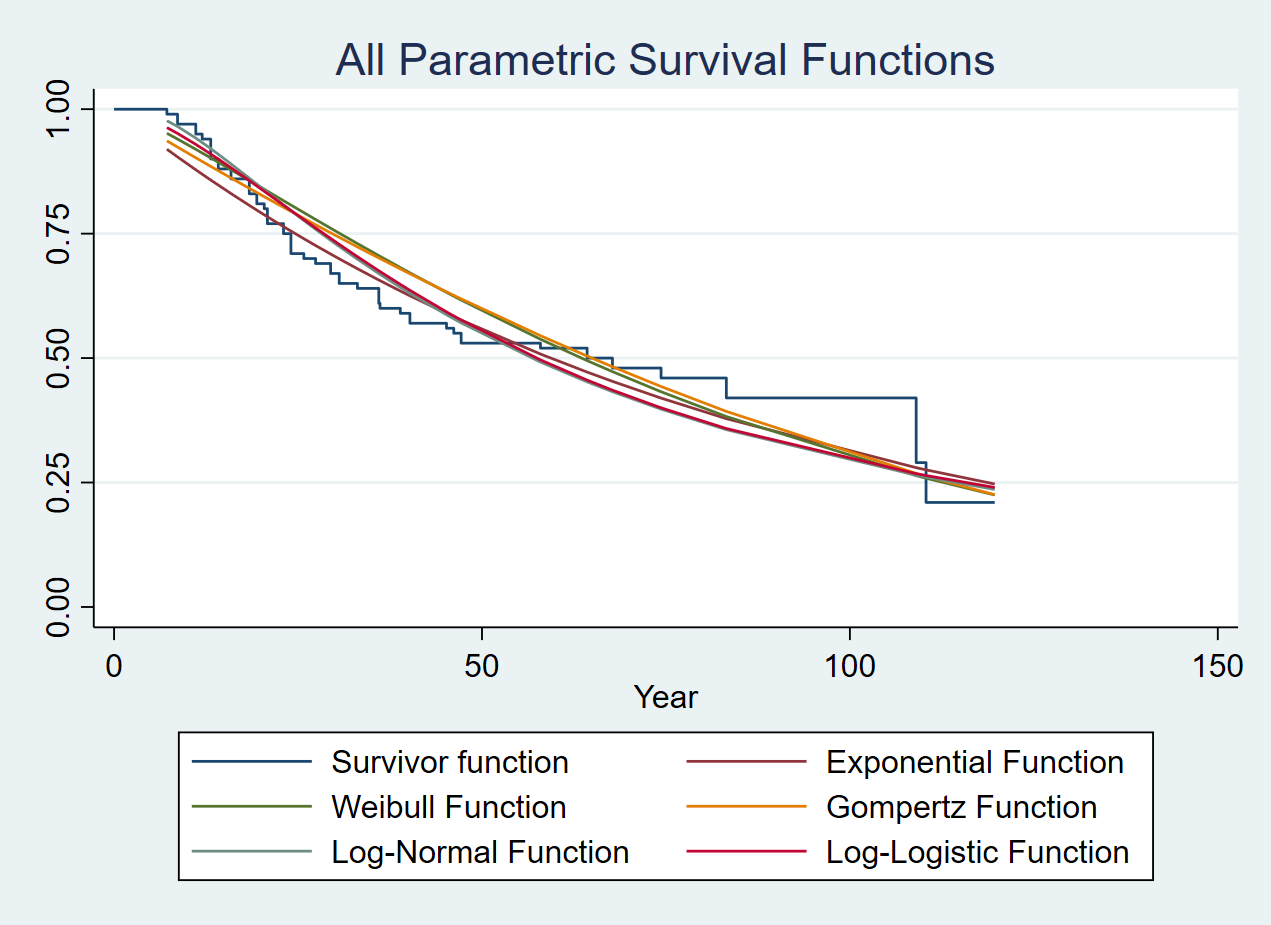


Proportion Surviving

Time (Month)

Parametric survival distribution of PET for time to progression

| Parameter | Exponential | Weibull | Gompertz | Log-normal | Log-logistic |
| --- | --- | --- | --- | --- | --- |
| Rate | -4.45 |  |  |  |  |
| Shape |  | -5.39 | -4.71 | 4.03 | 4.05 |
| Scale |  | 1.21 | 0.0052 |  | 0.64 |
| Location |  |  |  | 1.04 |  |
| AIC | 281.78 | 280.11 | 281.28 | 274.98 | 280.46 |
| BIC | 284.38 | 285.33 | 286.50 | 280.19 | 285.67 |

The Log-normal survival curves had the lowest AIC and BIC test statistics of PET, indicating that they best fit the observed data. Therefore, the decision-analytic model used a Log-normal survival curve of PET to estimate the time to progression.

Results of base-case deterministic analysis

| **Results** | | **Surgery + ET** | **PET** |
| --- | --- | --- | --- |
| Expected cost (£) | | £20,372.84 | £16,750.92 |
| Expected QALYs | | 17.78 | 17.15 |
| Incremental cost (£) | | £3,621.92 | - |
| Incremental QALYs | | 0.64 | - |
| ICER | | £5703.73 per QALY gain | - |
| Net monetary benefit (£) | λ=20000 | £335,314.10 | £326,235.84 |
|  | λ=30000 | £513,157.58 | £497,729.23 |
| Incremental net monetary benefit (£) | λ=20000 | £9,078.26 |  |
|  | λ=30000 | £15,428.35 |  |

**Reference**

1. Ishak, K.J., et al., *Overview of parametric survival analysis for health-economic applications.* Pharmacoeconomics, 2013. **31**(8): p. 663-75.

2. Latimer, N.R., *Survival analysis for economic evaluations alongside clinical trials—extrapolation with patient-level data: inconsistencies, limitations, and a practical guide.* Medical Decision Making, 2013. **33**(6): p. 743-754.

3. Chakrabarti, J., et al., *A randomised trial of mastectomy only versus tamoxifen for treating elderly patients with operable primary breast cancer-final results at 20-year follow-up.* Crit Rev Oncol Hematol, 2011. **78**(3): p. 260-4.

4. Gannon, M., et al., *National Audit of Breast Cancer in Older Patients: 2022 Annual Report*. 2022, The Royal College of Surgeons p. 94.

5. Johnston, S.J., et al., *A randomised trial of primary tamoxifen versus mastectomy plus adjuvant tamoxifen in fit elderly women with invasive breast carcinoma of high oestrogen receptor content: long-term results at 20 years of follow-up.* Annals of Oncology, 2012. **23**(9): p. 2296-2300.
