# Supplementary material for: Primary endocrine therapy versus surgery plus endocrine therapy for early-stage breast cancer in older women without frailty: a cost-effectiveness and value of implementation analysis
Source: Health Econ Rev. 2025 Sep 30;15:75. doi: 10.1186/s13561-025-00668-x (PMC12487327; doi:10.1186/s13561-025-00668-x)
Supplement: Supplementary file 2 — Supplementary Material 2 [file 13561_2025_668_MOESM2_ESM.docx]

AdViSHE

Assessment of the Validation Status of Health-Economic decision models

AdViSHE is a questionnaire that modellers can complete to report on the efforts performed to improve the validation status of their health-economic (HE) decision model. It is not intended to replace validation by model users but rather to inform the direction of validation efforts and to provide a baseline for replication of the results. In addition to using it after a model is finished, the modellers can use AdViSHE to guide validation efforts during the modelling process.

The modellers are asked to comment on the validation efforts performed while building the underlying HE decision model and afterwards. Many of the questions simply refer to the model documentation. AdViSHE is divided into five parts, each covering an aspect of validation:

- Part A: Validation of the conceptual model (2 questions)
- Part B: Input data validation (2 questions)
- Part C: Validation of the computerized model (4 questions)
- Part D: Operational validation (4 questions)
- Part E: Other validation techniques (1 question)

No final validation score is calculated, as the assessment of the answers and the overall validation effort is left to the model users. It is assumed that the model has been built according to prevailing modelling and reporting guidelines. For instance, the model builders would presumably adhere to the ISPOR-SMDM^†^ Modeling Good Research Practices (Caro et al., 2010) and/or CHEERS^†^ Statement (Husereau et al., 2013). Some questions may not be applicable to a particular model. If this is the case, the model builder should take the opt-out option and provide a justification of why this item is not deemed applicable.

Part A: Validation of the conceptual model (2 questions)

Part A discusses techniques for validating the conceptual model. A conceptual model describes the underlying system (e.g., progression of disease) using a mathematical, logical, verbal, or graphical representation. Please indicate where the conceptual model and its underlying assumptions are described and justified.

| The model was conceptualised based on a systematic review summarising the model-based economic evaluations, and expert opinion. |
| --- |

| **A1/ Face validity testing (conceptual model):** Have experts been asked to judge the appropriateness of the conceptual model?  If yes, please provide information on the following aspects:   - Who are these experts? - What is your justification for considering them experts? - To what extent do they agree that the conceptual model is appropriate?   If no, please indicate why not. |
| --- |

| Yes, the development and evaluation of the conceptual model incorporated expert judgment to ensure its clinical relevance, structural validity, and alignment with real-world practices. Professor Kwok-Leung Cheung, an expert Breast Cancer Surgeon at the Royal Derby Hospital Centre, University of Nottingham, was engaged to assess the model’s appropriateness. Prof Kwok-Leung Cheng reviewed the model structure to ensure that the proposed health states and transitions were clinically relevant and appropriate. |
| --- |

| Aspects to judge include: appropriateness to represent the underlying clinical process/disease (disease stages, physiological processes, etc.); and appropriateness for economic evaluation (comparators, perspective, costs covered, etc.). |
| --- |

| **A2/ Cross validity testing (conceptual model):** Has this model been compared to other conceptual models found in the literature or clinical textbooks?  If yes, please indicate where this comparison is reported.  If no, please indicate why not. |
| --- |

| Yes, the proposed model structure was compared with other structures found by a (1) systematic review of cost-effectiveness analyses for older people with primary breast cancer and (2) a published cost-effectiveness analysis of surgery plus adjuvant endocrine therapy. The chosen health states (progression-free, progressed, dead) were found to be a consistent way to reflect the natural history of older people with primary breast cancer. |
| --- |

Part B: Input data validation (2 questions)

Part B discusses techniques to validate the data serving as input in the model. These techniques are applicable to all types of models commonly used in HE modelling.

Please indicate where the description and justification of the following aspects are given:

- search strategy;
- data sources, including descriptive statistics;
- reasons for inclusion of these data sources;
- reasons for exclusion of other available data sources;
- assumptions that have been made to assign values to parameters for which no data was available;
- distributions and parameters to represent uncertainty;
- data adjustments: mathematical transformations (e.g., logarithms, squares); treatment of outliers; treatment of missing data; data synthesis (indirect treatment comparison, network meta-analysis); calibration; etc.

| **Search Strategy**  The clinical effectiveness data for primary endocrine therapy (PET) were sourced from the randomized controlled trial (RCT) by Chakrabarti et al., which had a 20-year follow-up. The effectiveness of surgery plus endocrine therapy (surgery + ET) was derived from a Cochrane Review (Morgan et al.), with the search strategy adhering to PRISMA guidelines.  **Data Sources, Including Descriptive Statistics**  The model incorporates parameters such as transition probabilities, utilities, and costs. These parameters, along with their values, ranges (95% confidence intervals), distributions, and specific data sources, are clearly presented. The descriptive statistics and parameters for treatment effectiveness were obtained from the RCT by Chakrabarti et al., while cost data were derived from various sources, including NHS reference costs and the British National Formulary.  **Reasons for Inclusion of These Data Sources**  The Chakrabarti RCT was selected for PET due to its long follow-up period and its UK-focused population, making it highly relevant to the analysis. The study’s inclusion and exclusion criteria aligned well with the target population of older women with early-stage breast cancer. Since the surgery arm of the RCT included both patients receiving surgery plus ET and those receiving surgery alone, the Cochrane Review was used to estimate the effectiveness of surgery + ET by applying hazard ratios (HRs). Additionally, expert clinical input and prior studies (e.g., Holmes et al.) were used to inform the model structure.  The Utility Data were identified from a systematic review that measure the health utility values for older women with early-stage breast cancer using preference-based instruments (EQ-5D-3L UK Tariff which suggested by NICE). Cost and Resource use data were identified from NHS reference costs and the British National Formulary to reflect the routine practice in the UK.  **Reasons for Exclusion of Other Available Data Sources**  Other RCTs included in the Cochrane review had shorter follow-up periods, which led to their exclusion from this analysis. A longer follow-up was crucial for capturing lifetime survival data and reducing uncertainty in the model’s predictions.  **Assumptions to Assign Values to Parameters Without Data**  No assumptions were made for parameters without data. All model parameters were derived from existing literature.  **Distributions and Parameters to Represent Uncertainty**  The model accounted for uncertainty through probabilistic sensitivity analysis (PSA), running 10,000 iterations using Monte Carlo simulations. Distributions (lognormal, beta, gamma) and parameters (mean, SD, α/β) are specified for probabilistic sensitivity analysis. Survival functions were selected via AIC/BIC. The results were presented using cost-effectiveness planes and cost-effectiveness acceptability curves (CEAC).  **Data Adjustments**  Kaplan-Meier curves digitized and reconstructed using Guyot et al.’s method. Surgery + ET effectiveness derived by applying HRs from the Cochrane Review to PET survival curves. There were no outliers or missing data because individual participant data were not used. There were no indirect treatment comparisons because treatment effects were obtained from a published systematic review and meta-analysis. |
| --- |

| **B1/ Face validity testing (input data):** Have experts been asked to judge the appropriateness of the input data?  If yes, please provide information on the following aspects:   - Who are these experts? - What is your justification for considering them experts? - To what extent do they agree that appropriate data has been used?   If no, please indicate why not. |
| --- |

| **Have Experts Been Asked to Judge the Appropriateness of the Input Data?**  Yes  **Who Are These Experts?**  Dr Sean Gavan (Senior Lecturer in Health Economics, Manchester Centre for Health Economics, The University of Manchester) was responsible for reviewing the model and survival analysis regression code iteratively.  **Justification for Considering Them Experts**  Dr. Sean Gavan, a Senior Lecturer in Health Economics at the University of Manchester, is a recognized expert due to his leadership in health economics research, specialization in survival analysis and cost-effectiveness modeling, and affiliation with the Manchester Centre for Health Economics. His peer-reviewed publications, grant-funded projects, and role in teaching advanced methods validate his authority to iteratively assess the model’s design and survival analysis code, ensuring alignment with rigorous health economic standards.  **Extent of Agreement on Data Appropriateness**  The expert agree that the data used are appropriate, supported by rigorous methodologies and relevance to the UK context. Clinical effectiveness for PET was sourced from Chakrabarti et al.’s RCT, selected for its 20-year follow-up and alignment with the target population, while surgery + ET effectiveness was derived from a Cochrane Review adhering to PRISMA guidelines. UK-specific utility (EQ-5D-3L) and cost data (NHS, BNF) ensured generalisability. Parameters were explicitly sourced from peer-reviewed studies, with no assumptions for missing data. Uncertainty was addressed via probabilistic sensitivity analysis (10,000 Monte Carlo iterations) using lognormal, beta, and gamma distributions. Adjustments included reconstructing Kaplan-Meier curves (Guyot et al.) and applying hazard ratios from the Cochrane Review. Exclusion of shorter-follow-up RCTs was justified to reduce extrapolation uncertainty. While limitations like RCT-real-world disparities (e.g., comorbidity differences) were acknowledged, the experts endorsed the data’s validity through transparency and sensitivity analyses confirming robustness. |
| --- |

| Aspects to judge may include but are not limited to: potential for bias; generalizability to the target population; availability of alternative data sources; any adjustments made to the data. |
| --- |

| **B2/ Model fit testing:** When input parameters are based on regression models, have statistical tests been performed?  If yes, please indicate where the description, the justification and the outcomes of these tests are reported.  If no, please indicate why not. |
| --- |

| **Has the Computerized Model Been Examined by Modelling Experts?**  Yes  **Who Are These Experts?**  Yubo Wang (First Author, Postgraduate Research Student, University of Manchester) and Dr. Sean Gavan (Research Fellow in Health Economics, University of Manchester).  **Justification for Considering Them Experts**  Yubo Wang as the first author, he had the model development, conducted systematic reviews, performed survival analysis, and validated the model using digitizing Kaplan-Meier curves, and parametric survival analysis. Dr Sean Gavan as Yubo's supervisor review the model. The detailed information of Dr Sean Gavan see B1.  **Independence of Experts**  No independent expert testing the model input parameters and regression model.  **Where Are the Results of This Review Reported?**  Parametric survival functions (Exponential, Weibull, Gompertz, Log-normal, Log-logistic) were tested for PET and surgery + ET effectiveness. The selection was based on AIC/BIC values (lowest criteria) and visual inspection for clinical plausibility. All the results reported in the Supplementary material 1 Appendices including AIC and BIC values, as well as the re-plotted KM curves. |
| --- |

| Examples of regression models include but are not limited to: disease progression based on survival curves; risk profiles using regression analysis on a cohort; local cost estimates based on multi-level models; meta-regression; quality-of-life weights estimated using discrete choice analysis; mapping of disease-specific quality-of-life weights to utility values.  Examples of tests include but are not limited to: comparing model fit parameters (R^2^, Akaike information criterion (AIC), Bayesian information criterion (BIC)); comparing alternative model specifications (covariates, distributional assumptions); comparing alternative distributions for survival curves (Weibull, lognormal, logit); testing the numerical stability of the outcomes (sufficient number of iterations); testing the convergence of the regression model; visually testing model fit and/or regression residuals. |
| --- |

Part C: Validation of the computerized model (4 questions)

Part C discusses various techniques for validating the model as it is implemented in a software program. If there are any differences between the conceptual model (Part A) and the final computerized model, please indicate where these differences are reported and justified.

| No differences between the conceptual model and the final computerized model. |
| --- |

| **C1/ External review:** Has the computerized model been examined by modelling experts?  If yes, please provide information on the following aspects:   - Who are these experts? - What is your justification for considering them experts? - Can these experts be qualified as independent? - Please indicate where the results of this review are reported, including a discussion of any unresolved issues.   If no, please indicate why not. |
| --- |

| The model was not reviewed by external experts. |
| --- |

| Aspects to judge may include but are not limited to: absence of apparent bugs; logical code structure optimized for speed and accuracy; appropriate translation of the conceptual model. |
| --- |

| **C2/ Extreme value testing:** Has the model been run for specific, extreme sets of parameter values in order to detect any coding errors?  If yes, please indicate where these tests and their outcomes are reported.  If no, please indicate why not. |
| --- |

| Yes  **Where are these tests and their outcomes reported?**  We conducted extreme value testing to check for potential coding errors by assigning specific, extreme values to key parameters. For example, we verified that setting cost parameters to zero resulted in a total cost of zero, and confirmed that discounted outcomes were consistently lower than undiscounted ones. These systematic internal checks helped ensure model robustness. However, the outcomes of these tests were not formally documented or reported in the manuscript. |
| --- |

| Examples include but are not limited to: zero and extremely high (background) mortality; extremely beneficial, extremely detrimental, or no treatment effect; zero or extremely high treatment or healthcare costs. |
| --- |

| **C3/ Testing of traces:** Have patients been tracked through the model to determine whether its logic is correct?  If yes, please indicate where these tests and their outcomes are reported.  If no, please indicate why not. |
| --- |

| **Have patients been tracked through the model to determine whether its logic is correct?**  Yes, patients were tracked through the model to ensure its logic was correct. For each cycle, we ensured that the total number of patients in each health state summed to the total cohort. This validation process was crucial to maintain the integrity of the model and ensure that it accurately reflected the expected transitions between health states.  **If no, please indicate why not.** |
| --- |

| In cohort models, this would involve listing the number of patients in each disease stage at one, several, or all time points (e.g., Markov traces). In individual patient simulation models, this would involve following several patients throughout their natural disease progression. |
| --- |

| **C4/ Unit testing:** Have individual sub-modules of the computerized model been tested?  If yes, please provide information on the following aspects:   - Was a protocol that describes the tests, criteria, and acceptance norms defined beforehand? - Please indicate where these tests and their outcomes are reported.   If no, please indicate why not. |
| --- |

| **Have individual sub-modules of the computerized model been tested?**  No  **If no, please indicate why not.**  Although individual sub-modules of the model were not tested separately, we ensured that the probabilistic switching function operated as intended, meaning that all parameters were treated probabilistically rather than being set to fixed values during probabilistic sensitivity analyses. This validation process was essential to confirm the overall accuracy and reliability of the model's logic. This approach helped maintain the integrity of the model's outcomes while accounting for uncertainties in the input parameters. |
| --- |

| Examples include but are not limited to: turning sub-modules of the program on and off; altering global parameters; testing messages (e.g., warning against illegal or illogical inputs), drop-down menus, named areas, switches, labelling, formulas and macros; removing redundant elements. |
| --- |

Part D: Operational validation (4 questions)

Part D discusses techniques used to validate the model outcomes.

| **D1/ Face validity testing (model outcomes):** Have experts been asked to judge the appropriateness of the model outcomes?  If yes, please provide information on the following aspects:   - Who are these experts? - What is your justification for considering them experts? - To what extent did they conclude that the model outcomes are reasonable?   If no, please indicate why not. |
| --- |

| **Have experts been asked to judge the appropriateness of the model outcomes?**  Yes, Prof Kwok-Leung Cheng (Breast Cancer Surgeon at the Royal Derby Hospital Centre, University of Nottingham) reviewed the model outputs in discussion with the lead author to confirm that the findings were applicable to real-world clinical settings and aligned with expected clinical practice.  **If no, please indicate why not.** |
| --- |

| Outcomes may include but are not limited to: (quality-adjusted) life years; deaths; hospitalizations; total costs. |
| --- |

| **D2/ Cross validation testing (model outcomes):** Have the model outcomes been compared to the outcomes of other models that address similar problems?  If yes, please provide information on the following aspects:   - Are these comparisons based on published outcomes only, or did you have access to the alternative model? - Can the differences in outcomes between your model and other models be explained? - Please indicate where this comparison is reported, including a discussion of the comparability with your model.   If no, please indicate why not. |
| --- |

| **Have the model outcomes been compared to the outcomes of other models that address similar problems?**  Yes  **Provide information on the following aspects:**  The results were compared to those reported by Holmes et al., who also conducted a model-based health economic evaluation of surgery with adjuvant ET compared with PET for older women.  **Where is this comparison reported?**  The comparison is discussed in the "Discussion" section, where the present study's results align with Holmes et al. despite differences in cost estimates. |
| --- |

| Other models may include models that describe the same disease, the same intervention, and/or the same population. |
| --- |

| **D3/ Validation against outcomes using alternative input data:** Have the model outcomes been compared to the outcomes obtained when using alternative input data?  If yes, please indicate where these tests and their outcomes are reported.  If no, please indicate why not. |
| --- |

| Yes  **Where are these tests and their outcomes reported?**  Sensitivity analyses using alternative RCTs, such as those by Johnston et al., are mentioned in the "Discussion" section, and |
| --- |

| Alternative input data can be obtained by using different literature sources or datasets, but can also be constructed by splitting the original data set in two parts, and using one part to calculate the model outcomes and the other part to validate against. |
| --- |

| **D4/ Validation against empirical data:** Have the model outcomes been compared to empirical data?  If yes, please provide information on the following aspects:   - Are these comparisons based on summary statistics, or patient-level datasets? - Have you been able to explain any difference between the model outcomes and empirical data? - Please indicate where this comparison is reported.   If no, please indicate why not.  **D4.A/** Comparison against the data sources on which the model is based (dependent validation). |
| --- |

| Yes, the simulated overall survival estimates were compared with those observed in a published national patient-level observational cancer registry study, detailed information in the Discussion Section.  **If no, please indicate why not.** |
| --- |

| **D4.B/** Comparison against a data source that was not used to build the model (independent validation). |
| --- |

| No |
| --- |

Part E: Other validation techniques (1 question)

| **E1/ Other validation techniques:** Have any other validation techniques been performed?  If yes, indicate where the application and outcomes are reported, or else provide a short summary here. |
| --- |

| No other validation techniques was used. |
| --- |

| Examples of other validation techniques: structured “walk-throughs” (guiding others through the conceptual model or computerized program step-by-step); naïve benchmarking (“back-of-the-envelope” calculations); heterogeneity tests; double programming (two model developers program components independently and/or the model is programmed in two different software packages to determine if the same results are obtained). |
| --- |
